# Biomarkers in Adult Dermatomyositis: Tools to Help the Diagnosis and Predict the Clinical Outcome

**DOI:** 10.1155/2019/9141420

**Published:** 2019-01-14

**Authors:** Charles Cassius, Hélène Le Buanec, Jean-David Bouaziz, Reyhan Amode

**Affiliations:** ^1^Dermatology Department, Hôpital Saint-Louis, Assistance Publique-Hôpitaux de Paris (AP-HP), Paris, France; ^2^INSERM U976, Laboratory of Oncodermatology, Immunology, and Cutaneous Stem Cells, Hôpital Saint-Louis, Paris, France; ^3^Université Paris Diderot-Paris VII, Sorbonne Paris Cité, Paris, France; ^4^Catholic University of Louvain, CHU UCL Namur, Yvoir, Belgium; ^5^Dermatology Department, Hôpital Bichat Claude Bernard, Assistance Publique-Hôpitaux de Paris (AP-HP), Paris, France

## Abstract

Dermatomyositis pathophysiology is complex. In recent years, medical research has identified molecules associated with disease activity. Besides providing insights into the driving mechanisms of dermatomyositis, these findings could provide potential biomarkers. Activity markers can be used to monitor disease activity in clinical trials and may also be useful in daily practice. This article reviews molecules that could be used as biomarkers for diagnosis and monitoring dermatomyositis disease activity.

## 1. Introduction

Dermatomyositis (DM) is a rare and chronic acquired autoimmune disorder that affects primarily the skin, muscles, and lungs. It belongs to the spectrum of the idiopathic inflammatory myopathies (IIMs) with polymyositis (PM).

Skin lesions in DM play an important role in the diagnosis. Indeed, in more than half of the patients, they precede muscle involvement by months or years. The skin involvement in DM is characterized by seven types: pathognomonic (Gottron's sign and Gottron's papules), characteristic (periorbital erythema, periungual telangiectasia, shawl, and V-sign), compatible (poikiloderma, holster sign, and periorbital edema), less common (necrotic or vesiculobullous lesions and calcinosis cutis), rare (mechanic's hand, flagellate erythema, and panniculitis), and nonspecific (Raynaud's phenomenon, pruritus, and photosensitivity) skin manifestations.

DM is strongly associated with a wide range of cancers. Cancers are detected for most of them within 1 year after the diagnosis of myositis, which is considered as paraneoplastic.

Although autoantibodies and particularly myositis-specific antibodies (MSAs) are one of the most important biomarkers in DM, other molecules or parameters (i.e., proteins, cytokines, chemokines, and classic blood laboratory tests) have been extensively studied and many of them could be used to follow disease activity or predict patients' prognosis.

The purpose of this article is to review the candidate biomarkers that could be used for the diagnostic, prognostic, or therapeutic approach in DM.

## 2. Material and Methods

### 2.1. Data Sources

We identified relevant studies on biomarkers associated with DM published before May 1, 2018 that were listed in the following international scientific databases: MEDLINE and the Cochrane Library. Searches were restricted to articles written in English. Relevant references cited in the original articles were also reviewed. We also hand-searched additional relevant studies.

The following search strategy was applied in MEDLINE:

1. “Dermatomyositis” [title/abstract] or “DM” [title/abstract]
2. “Neoplasm” [title/abstract] or “paraneoplastic” [title/abstract] or “cancer” [title/abstract] or “interstitial lung disease” [title/abstract] or “lung disease” [title/abstract] or “mortality” [title/abstract] or “prognosis” [title/abstract] or “biomarker” [title/abstract] or “ferritin” [title/abstract] or “treatment response” [title/abstract] or “heart disease” [title/abstract] or “relapse” [title/abstract] or “toxicity” [title/abstract]
3. 1 and 2

### 2.2. Study Selection and Data Extraction

Studies had to meet the following eligibility criteria: (1) Studies included cases in accordance with a probable or definitive diagnosis of DM based on the criteria of Bohan and Peter [1] and the European Neuromuscular Center (ENMC) [2]or a diagnosis of clinical amyopathic DM (CADM) based on Sontheimer [3]. (2) Studies compared DM with at least one healthy donor (HD) control group. Studies were excluded if (1) they were expert opinions, case reports, or letters that were not published in full and (2) the extraction of relevant data was impossible.

## 3. Results

### 3.1. Antibodies

Autoantibodies (Ab) found during DM are classified into myositis-specific antibodies (MSAs) and myositis-associated antibodies (MAAs). MSAs are detected in between 30% and 50% of patients with DM [4, 5]. The presence of more than one MSA in each patient is uncommon.

**Figure 1:**
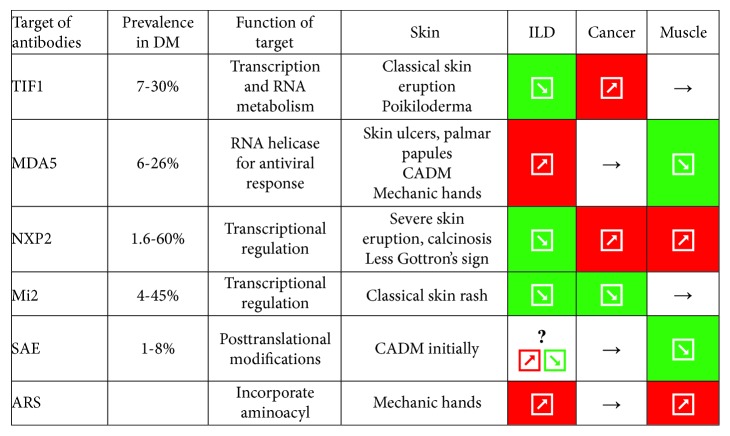
Targets, prevalence, and clinical association of myositis-specific antibodies. ILD: interstitial lung disease; TIF: transcription intermediary factor; RNA: ribonucleic acid; MDA: melanoma differentiation-associated gene; CADM: clinical amyopathic dermatomyositis; NXP: nuclear matrix protein; SAE: small ubiquitin-like modifier-activating enzyme; ARS: aminoacyl tRNA synthetase.

**Figure 2:**
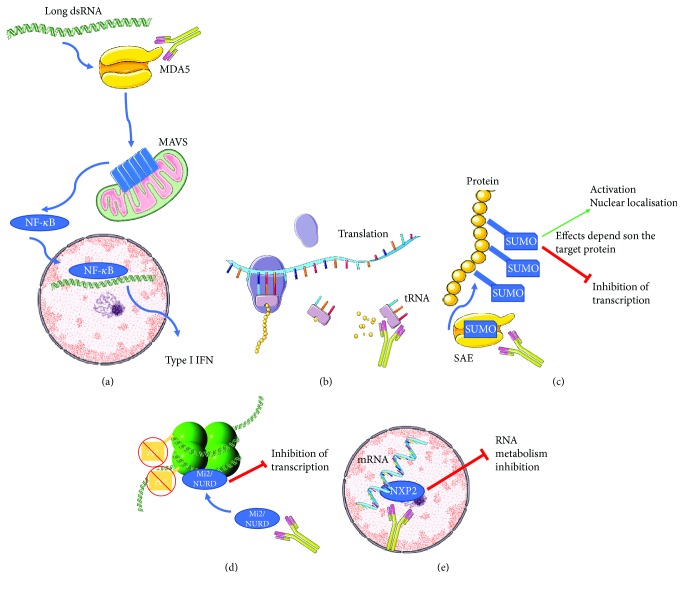
Targets and the simplified downstream effects of their autoantibodies encountered during dermatomyositis. (a) MDA5 recognizes the long double-strand RNA leading through the RIG1-MDA5 pathway to the cleavage of the mitochondrial antiviral signaling protein (MAVS), the nuclear translocation of NF-*κ*B, and the production of type I interferon. (b) Transfer RNA (tRNA) (Jo-1, PL-7, PL-12, EJ, OJ, KS, Zo, and Ha) helps the ribosome-recruiting aminoacyl to the site of translation. (c) Small ubiquitin-like modifier-activating enzymes (SAEs) are members of an enzyme complex leading to the SUMOylation of targeted proteins leading to either their translocation to the nucleus or the inhibition of transcription. (d) Mi-2 is a component of the nucleosome remodeling deacetylase complex (NuRD) which actively deacetylates histones leading to the compaction of chromatin and subsequently to the inhibition of transcription. (e) The nuclear matrix protein 2, which localizes in the promyelocytic leukemia (PML) nuclear bodies, binds to mRNA in the nucleus and can subsequently lead to RNA metabolism inhibition. Transcription intermediary factor family antigens have been deliberately omitted in this figure due to their broad mechanism of action.

The question why antigens are targeted only in DM and not during other autoimmune diseases, such as systemic lupus erythematosus, is still unanswered. The relation between DM-specific autoantigens' targets is emerging, suggesting a common pathway implicated in chromatin modulation. For example, TIF1-*β* binds to Mi-2*α*, a member of the NuRD complex, and TIF1-*β* is heavily sumoylated, a process which involves the small ubiquitin-like modifier-activating enzyme (SAE) complex. The localization of NXP-2 to PML is dependent on a sumoylation which involves the SAE complex. There are connections between these autoantigens' targets which may contribute to their immunologic targeting in DM patients. Clinical implications and biological functions are resumed in Figures 1 and 2.

#### 3.1.1. Anti-TIF1-*γ*/*α* and *β* (Formerly Anti-155/140) Ab

TIF1-*α* (TRIM 24), TIF1-*β* (TRIM 28), and TIF1-*γ* (TRIM 33) belong to the TIF (transcription intermediary factor) family of transcription cofactors which is implicated in the signalization pathway of transforming growth factor- (TGF-) *β* [6].

Anti-TIF1-*γ*/*α* and *β* are not only clinically associated with a low prevalence of fever, arthritis, ILD, or mechanic's hand but are also clinically associated with a higher prevalence of classical skin eruption (Gottron's papules, trunk erythema, V-sign, and shawl sign) with poikiloderma and malignancy. The prevalence of anti-TIF1-*γ*/*α* in DM was 7–30% [4, 7–9] and 22–100% in cancer-associated DM. The prevalence of malignancy in anti-TIF1-*γ*/*α*-Ab-positive patients was 42–100%. In a recent Chinese longitudinal cohort, anti-TIF1-*γ* Ab was associated with an increased risk of cancer compared to the general population (standardized incidence ratio = 17.28, 95% CI (11.94–24.24)) [10]. The sensitivity and specificity of anti-TIF1-*γ*/*α* for cancer-associated DM were 78% and 79%, respectively [11]. Cancer is frequently detected within 1 year of myositis diagnosis [12]. The association between anti-TIF1-*γ*/*α* and cancer does not seem to apply to young adults [13] and was not found in several studies [4].

#### 3.1.2. Anti MDA5 (Formerly Anti-CADM 140) Ab

Anti-CADM 140 was described in 2005 in Japanese patients with clinically amyopathic dermatomyositis. This Ab was significantly associated with rapidly progressive interstitial lung disease (RP-ILD) compared to anti-CADM 140 negative dermatomyositis [14]. The targeted antigen was then identified as melanoma differentiation-associated gene 5 (MDA5), which belongs to the RIG-I-like receptors. These cytosolic pattern recognition receptors detect viral RNA and initiate innate immune response.

Reported anti-MDA5 Ab prevalence among DM patients varies from 6 to 26% [15–17].

Several studies confirmed the negative prognostic value of anti-MDA5 auto-Ab in Asian patients, related to RP-ILD [18–21]. Small cohorts suggested that the MDA5 titer could predict relapses or treatment outcome [22, 23].

Discordant results were found in other populations, with an inconstant association with severe lung disease [4, 16, 24–26].

Finally, a systematic meta-analysis found pooled sensitivity, specificity, and AUC values of 0.83 (95% CI: 0.77–0.88), 0.86 (95% CI: 0.80–0.91), and 0.87 (95% CI: 0.84–0.90) for anti-MDA5 Ab in DM with RPILD versus without RPILD [25].

With regard to skin presentation, anti-MDA5 Ab has been linked to skin ulcers, palmar papule occurrence, and mechanic's hands [4, 18, 27].

#### 3.1.3. Anti-NXP-2 (Formerly Anti-MJ) Ab

Anti-NXP-2 Ab, also known as anti-MJ, recognizes the nuclear matrix protein 2 (NXP-2; also known as MORC3), which plays an important role in diverse nuclear functions such as RNA metabolism and maintenance of nuclear architecture. They contain three conserved domains, including (i) a GHL-ATPase domain at the N terminus, (ii) a CW-type zinc finger domain containing four conserved cysteine and two tryptophan residues in the middle portion, and (iii) a coiled-coil dimerization domain at the C terminus. NXP-2 localizes in the promyelocytic leukemia (PML) nuclear bodies, where it recruits and activates p53 to induce cellular senescence [28]. Anti-NXP-2 Ab was originally described in juvenile DM [29].

The prevalence of anti-NXP-2 in adult DM was 1.6–30% [4, 7, 30, 31]. Anti-NXP-2 seems to be associated with severe classical skin rash and calcinosis, less Gottron's sign and Gottron's papules [4, 32], more dysphagia and muscle weakness, and less ILD [33]. Data concerning association with cancer are controversial [10, 31, 33, 34], but several studies showed a greater risk of neoplasm. Anti-NXP-2 serum level was found correlated with physician global assessment (PGA) VAS and muscle VAS. Moreover, in a longitudinal analysis, anti-NXP-2 decreased and even disappeared during clinical remission and was correlated with PGA VAS, constitutional VAS, muscle VAS, and cutaneous VAS [33].

#### 3.1.4. Anti-Mi-2 Ab

The targets of anti-Mi-2 Ab are components of the nucleosome remodeling deacetylase complex (NuRD). Anti-Mi-2 is not only associated with the classic skin features of DM (Gottron's papules, heliotrope rash, shawl sign, and V-sign) but it is also associated with a low risk of ILD [35] and cancer. The prevalence in DM was estimated at 4–45% [5, 8, 9, 35, 36]. Anti-Mi-2 Ab is associated with a good response to steroid therapy and a good prognosis.

#### 3.1.5. Anti-SAE Ab

The target antigens of anti-SAE Ab are the small ubiquitin-like modifier-activating enzyme A subunit (SAE1) and SUMO-1-activating enzyme B subunit (SAE2). These are enzymes involved in the posttranslational modification of specific proteins known as SUMOylation.

The prevalence of anti-SAE Ab is estimated at 1–8% [7, 37–41]. Anti-SAE was associated with initial CADM progressing to myositis associated with systemic features including dysphagia [37, 39]. There are conflicting data concerning ILD with high prevalence in Japanese [39] and low prevalence in Caucasian [37] patients, with nonsevere-ILD in any case. Anti-SAE was not associated with malignancies in most studies [7, 37–41], whereas a recent longitudinal cohort study showed an increased risk of cancer [10].

#### 3.1.6. Anti-Aminoacyl tRNA Synthetase (ARS) Ab

Anti-ARS Ab recognizes the cytoplasmic amino acid-charging enzymes, aminoacyl tRNA synthetases. Eight have been reported so far (Jo-1, PL-7, PL-12, EJ, OJ, KS, Zo, and Ha), and they are usually mutually exclusive.

Due to common clinical aspects, Targoff proposed a disease entity termed “anti-synthetase syndrome,” defined by myositis, ILD, fever, Raynaud's phenomenon, arthritis, and mechanic's hands [42]. Nevertheless, the distribution and timing of myositis, ILD, and rashes differ among patients with individual anti-ARS Abs.

Myositis is associated with anti-Jo-1, anti-EJ, and anti-PL-7. DM-specific skin manifestations (heliotrope rash and Gottron's sign) are preferentially observed in patients with anti-Jo-1, anti-EJ, anti-PL-7, and anti-PL-12. Most patients with anti-ARS Abs develop ILD if absent at disease onset [8].

Anti-ARS-Ab-positive ILD could be of better prognosis compared to other serotypes [43]. However, in a recent study focusing on antisynthetase syndrome in a Chinese cohort, anti-PL7 Ab was associated with RP-ILD [44]. Among anti-ARS Ab patients in IIM, anti-Jo1 could have a better survival rate [45].

Thus, ARS Ab are interesting biomarkers, defining subgroups of patients with specific presentations, organ involvements and prognosis.

***To conclude, MSAs are useful tools and they should be systematically searched at diagnosis as they all are associated with a peculiar clinical phenotype, visceral association, and neoplasm risk. Moreover, anti-MDA5 and anti-NXP-2 Ab titers seem to be associated with the clinical course of the disease.***

### 3.2. Cytokines

Cytokines are signaling molecules essential in the coordination of inflammatory responses. Produced by a wide variety of cells (immune system, endothelium, or epithelium cells) they mediate anti- or proinflammatory response. Numerous cytokines have been described to be elevated in serum or to be expressed in muscle tissue. Moreover, proinflammatory cytokines such as interleukin- (IL-) 1, IL-15, and tumor necrosis factor- (TNF-) *α* can, beneath their action on immune cells, affect muscle and skin cell metabolism and regeneration.

#### 3.2.1. IL-1 Cytokine Family: IL-18

IL-18 is a Th1 inflammatory cytokine belonging to the IL-1 cytokine family and shares structural similarities with IL-1*β*. It is mainly produced by antigen-presenting cells, including not only macrophages and dendritic cells but also keratinocytes [46]. It interacts with IL-12 to produce interferon- (IFN-) *γ*, induces both proliferation and differentiation of naive T cells, and exerts intrinsic attraction through its receptor (IL-18R) or through the stimulation of several chemokines, including monocyte chemoattractant protein- (MCP-) 1.

Tucci et al. [47] found IL-18 in the muscle biopsy specimen from patients with DM by immunohistochemistry and in situ hybridization, whereas it was absent in HD. Several studies [47–50] have found IL-18 to be elevated in the serum of DM or CADM compared to HD. IL-18 was correlated with ferritin [48] and clinical activity scores [49, 50]. Concerning interstitial lung disease (ILD), Gono et al. [48] and Yang et al. [49] found that IL-18 was higher in DM-associated ILD (DM-ILD) compared to DM without ILD but no difference was found between RP-ILD compared to non-RP-ILD [23, 51]. Zou et al. [52] confirmed these results in CADM neutrophils: they found an increase of IL-18 mRNA in CADM patients compared to classical DM (cDM) and HD.

IL-18 can also be used as a prognosis marker as Muro et al. [51] assessed that in RP-ILD, IL-18 serum level was lower in the survivor group compared to the nonsurvivor group. Similarly, Gono et al. [23] showed that IL-18 significantly decreased after treatment in survivors whereas it increased (not significantly) in nonsurvivors.

#### 3.2.2. IL-2 Cytokine Family: IL-15

IL-15, a member of the IL-2 family, is a proinflammatory cytokine with pleiotropic activity. IL-15 is constitutively expressed by many cell types, including monocytes, macrophages, dendritic cells (DCs), keratinocytes, and fibroblasts. It stimulates the proliferation and activation of macrophages, CD4 memory lymphocytes, and cytotoxic CD8 lymphocytes. It also stimulates the proliferation of myoblasts, endothelial cells, and many others [53].

Serum IL-15 is elevated in PM/DM compared to HD [54, 55] and seems to be higher in the active period of the disease [54]. In muscle biopsy specimens from patients with DM, IL-15 was strongly expressed and diminishes after treatment [56, 57].

*In vitro* IL-15 acts on skeletal muscles, increasing accumulation of contractile proteins in differentiated myocytes and muscle fibers and indicating a role for IL-15 in skeletal muscle fiber hypertrophy [58]. Cultured human myoblasts from patients with myositis constitutively produce a low level of cytoplasmic and secreted IL-15, which was also observed in healthy controls; however, mRNA and protein production was increased in DM patients by stimulation with IL-1*α*, IL-1*β*, TNF-*α*, or IFN-*γ* [56].

#### 3.2.3. IL-17 Cytokine Family: IL-17

IL-17 is a marker cytokine of Th17 cells. It is secreted by activated memory T cells in response to their stimulation with IL-23 and act as a proinflammatory mediator.

Expression of IL-17 has been detected in inflammatory infiltrates of DM muscle biopsies [59]. Although serum IL-17 level was not different between DM and HD, it was higher in early stage compared to established disease [60].

#### 3.2.4. IL-6/IL-12 Cytokine Family: IL6, IL-23, IL-27, and IL-35

IL-6 is a cytokine with redundant and pleiotropic activities. It contributes to host defense against infections and tissue injuries by inducing the acute-phase response and activating immune responses and hematopoiesis. It is secreted not only by neutrophils, monocytes, and macrophages but also by endothelial cells, mesenchymal cells, and fibroblasts. IL-6 promotes the maturation and survival of B cells and acts in combination with TGF-*β* in an imbalance toward Th17 response by CD4 T cells. IL-6 is also a major regulator of the initiation of acute-phase responses [61].

IL-6 serum level was found increased in DM compared to HD [62–64]. IL-6 serum level was higher in ILD compared to non-ILD [62] and also in RP-ILD compared to non-ILD [65] but not in RP-ILD compared to chronic-ILD (C-ILD) [66]. IL-6 was positively correlated with global VAS score [63, 65], ferritin, and C-reactive protein (CRP) [64, 65], and there was a correlation between the change in IL-6 level between first and second visit and the change of the physician global assessment [67]. Concerning survival, IL-6 was significantly higher in nonsurvivors compared to survivors. The cumulative survival rate was lower in patients with serum IL-6 levels > 9 pg/mL than in patients with serum IL-6 levels < 9 pg/mL. However, serum IL-6 was not a significant prognostic factor in multiple regression analysis [68].

IL-23 is a member of the IL-12 family with IL-12, IL-23, and IL-27. It facilitates the expansion and maintenance of Th17 cells. IL-23 is mainly expressed by macrophages and dendritic cells. IL-23R is found in memory T cells, NKT cells, macrophages, dendritic cells, and naive T cells upon activation by TGF-*β* and IL-6.

The serum level of IL-23 was found to be significantly higher in DM patients, and like IL-17, it was higher in early stage compared to established disease [60].

IL-27 is mainly produced by antigen-presenting cells and plays a key role in regulating T cell differentiation and function with a dual action reported: a proinflammatory action associated with Th1 polarization, and an anti-inflammatory action stimulating IL-10 production, survival of Tregs, and expression of inhibitory receptors on T cells [69].

IL-27 serum level was found elevated in DM compared to HD [50, 70]. Considering clinical (ILD vs. non-ILD) and biological (creatine kinase (CK) low vs. CK high) subgroups, IL-27 was elevated only in ILD and “CK high” groups [50]. IL-27 was not correlated with the global VAS score [50] and did not changed after treatment [70].

IL-35 is secreted in response to IFN-*γ* and agonists of toll-like receptor 3 (TLR3) and TLR4 by a wide range of regulatory lymphocytes. In contrast to the proinflammatory effect of other cytokines of the IL-12 family (IL-12 and IL-23), IL-35 potently inhibits the CD4+ effector T cells including Th1 and Th17 cells through the expansion of Treg cells and IL-10 production. IL-35 has been studied in several autoimmune diseases, but its role remains controversial [71].

Serum level of IL-35 was found to be elevated in DM [72, 73]. A positive correlation between the serum level of IL-35 and ferritin [73], MYOACT, and PGA score [72] has been demonstrated in DM. Mann et al. [72] studied the expression of IL-35 in muscle biopsy specimens of 9 DM and 10 PM patients and found an overexpression of IL-35 compared to those of HD patients.

#### 3.2.5. TNF Cytokine Family: TNF-*α*, LIGHT, BAFF, and APRIL

TNF-*α* is a proinflammatory cytokine that is produced not only by macrophages but also by neutrophils, monocytes, T cells, or natural killer (NK) cells. The production of TNF-*α* is stimulated by IL-1, IFN, and granulocyte macrophage-colony stimulating factor (GM-CSF) and inhibited by IL-6. It is a pleiotropic cytokine that causes cytolysis of many cell types, induces synthesis of IL-1, and stimulates phagocytosis and expression of major histocompatibility complex (MHC) I and II on lymphocytes. The pathophysiological role of TNF-*α* in the muscle tissue of myositis patients have been reviewed in Reference [74].

Gono et al. showed that TNF-*α* is higher in ILD-DM than in no-ILD-DM and that the high serum TNF-*α* level was correlated with global disease activity in DM [62].

LIGHT (which stands for homologous to Lymphotoxin, exhibits Inducible expression and competes with HSV Glycoprotein D for binding to Herpesvirus entry mediator, a receptor expressed on T lymphocytes), a member of the TNF family, can activate CD4+ and CD8+ T cells, monocytes and macrophages, natural killer cells, immature dendritic cells, and platelets. LIGHT, which is expressed in immature DCs and activated T cells, signals through TNFRSF14 (also known as HVEM) inducing the activation of CD8+ T cells. The serum LIGHT has been reported as a potential biomarker in rheumatoid arthritis, inflammatory bowel disease, or atopic dermatitis.

The LIGHT serum level was found elevated in DM-ILD compared to non-ILD-DM and HD; it was not elevated in non-ILD compared to HD. In the ILD subgroup, LIGHT was more elevated in RP-ILD than in C-ILD. The serum LIGHT level was not different between survivors and nonsurvivors in the ILD group, but LIGHT was correlated with a diffusing capacity of the lung for carbon monoxide (DLCO) and with the ground glass opacity score [66].

The B cell-activating factor (BAFF) and proliferation-inducing ligand (APRIL) are two TNF family members. They are primarily expressed by monocytes, macrophages, dendritic cells, neutrophils, and mast cells, and both play a key role in B-cell survival, activation, isotype switching, and T-independent Ab responses [75]. Furthermore, recent studies have shown a role of BAFF as costimulatory molecule of both CD4+ and CD8+ T cells [76].

The BAFF level was elevated in DM compared to HD both at the protein [77, 78] and mRNA levels [79]. BAFF was mildly correlated with global, muscle, and cutaneous disease activity scores (myositis disease activity assessment tool), and it decreased after treatment. The expression of BAFF was also studied in the muscle in 9 DM as compared to 4 HD by reverse transcription and polymerase chain reaction (RT-PCR) and immunohistochemistry [80] showing an increase in BAFF expression and the coexpression in injured muscle of BAFF-R and CD4+ and CD19+.

APRIL was neither elevated at the protein nor mRNA level [77, 78].

#### 3.2.6. Miscellaneous Cytokines: HMGB1

The high-mobility group protein 1 (HMGB1) protein belongs to the family of damage-associated molecular patterns, which includes ligands of pattern recognition receptors, especially via TLR4 expressed in skeletal muscle fibers. HMGB1 is among the most important chromatin proteins; it can be released by necrotic cells and rapidly moved from the nucleus into the cytoplasm and circulation during inflammation. It promotes the release of cytokines and attracts inflammatory cells. Increased serum and/or plasma levels of HMGB1 have been reported in autoimmune diseases such as systemic lupus erythematosus (SLE) [81, 82].

In a study conducted by Shu et al. [81], HMGB1 levels in DM were significantly higher than those in HD. The levels of HMGB1 in PM/DM patients with ILD were higher than in patients without ILD. In a 1200-week survival analysis, patients with higher HMGB1 had significantly worse prognosis. This was confirmed by a multivariate Cox proportional hazard model evaluation of mortality (HR = 2.10, *p* = 0.023).

#### 3.2.7. Type I IFNs

Type I IFNs (IFN-*α*, IFN-*β*, IFN-*ω*, IFN-*ε*, and IFN-*κ*) activate intracellular antimicrobial programs and influence the development of innate and adaptive immune responses. Type I interferons activate the JAK-STAT pathway leading to the transcription of IFN-stimulated genes (ISGs). They are protective in acute viral infection and can be deleterious in autoimmune diseases.

A marked upregulation of downstream biomarkers of this pathway has been found in the muscle, skin, and blood of patients with DM [83–89].

In a group of 56 adult and juvenile patients, disease activity correlated significantly with the type I IFN gene signature (IFIT1, G1P2, and IRF7). Type I IFN-induced chemokine serum levels (IFN-inducible T cell *α* chemoattractant, IFN-*γ*-inducible 10 kDa protein, monocyte chemotactic protein 1 (MCP-1), and MCP-2) corroborated this finding [63]. The magnitude of the overexpression of IFN-inducible genes correlated with disease activity in Walsh et al.'s cohort [85].

Cutaneous activity through the cutaneous disease severity index (CDASI) has also been linked to blood type I IFN gene signature. Both IFN-*α* and IFN-*β* correlated with type I IFN gene signature but only the IFN-*β* protein level was significantly elevated [90].

Horai et al. showed significantly higher IFN-*α* serum level in anti-MDA5-Ab-positive patients compared to other serotypes. All the anti-MDA5-A-positive patients presented RP-ILD [91]. The study of Sun et al. also evidenced a high IFN-*α* level in CADM in the ILD subgroup of patients [92]. These data suggest that IFN-*α* might play a role in inflammation triggered in ILD.

***To conclude, many cytokines seem to play a pivotal role in the pathogenesis of DM. However, many of them cannot yet be used as an easy biomarker because they seem to be implicated at the tissue level and are therefore difficult to detect in everyday practice or they are related to nonspecific inflammation or both (IL-15, IL-17, IL-23, IL-27, and TNF-α). Some results are encouraging but need to be validated in larger studies (LIGHT and HMGB1).***

***IL-35, type I IFN, and BAFF are both elevated during DM, as validated by several studies and correlated with clinical scores.***

***IL-18 and IL-6 have been studied in several studies and are higher in DM and ILD and correlated with clinical scores. Moreover, IL-18 and IL-6 could be used as prognosis markers for treatment response and mortality, respectively.***

### 3.3. Chemokines

Chemokines are small cytokines known for their ability to induce the migration of cells such as lymphocytes, dendritic cells, macrophages, and stem cells. Based on the cellular context and the site of expression, chemokines can be divided into “inflammatory chemokines”—those that are synthesized and promote recruitment of cells during inflammation and “homeostatic chemokines”—those that are constitutively expressed in specific tissues where they regulate leukocyte homing. Some chemokines participate both in immune defense during inflammation and in physiological trafficking of resting leukocytes.

#### 3.3.1. IL-8 or CXCL8

IL-8 (CXCL8) is a proinflammatory chemokine. It signals via binding with CXCR1 (IL-8R*α*) or CXCR2 (IL-8R*β*). Macrophages and both epithelial and endothelial cells produce CXCL8 in response to infection or injury. The first function of CXCL8 is to induce the chemotaxis of granulocytes and lymphocytes. The second function of CXCL8 is to activate the angiogenic response. CXCL8 signaling in vascular endothelial cells induces cell proliferation, survival, and migration [93].

CXCL8 was found elevated in DM and CADM associated with anti-MDA5 or anti-ARS Ab compared to HD in one study [62]. CXCL8 seems to be implicated in CADM and ILD since serum level was increased in CADM compared to that in classical DM with ILD and HD [52] and in RP-ILD but not in C-ILD compared to non-ILD [65] or in ILD compared to non-ILD in anti-MDA5 or anti-ARS-Ab-positive patients [62]; however, in this last study, in multiple linear regression analysis CXCL8 was not associated with ILD. A positive correlation was found between CXCL8 and MITAX [62], global VAS, pulmonary VAS, and serum ferritin level [65].

#### 3.3.2. CX3CL1

CX3CL1 exhibits chemotactic activity for monocytes, T cells, NK cells, dendritic cells, and B cells. CX3CL1 can be expressed by many cell types of hematopoietic or nonhematopoietic origin. CX3CR1, the receptor of CX3CL1, is expressed in NK cells, CD14+ monocytes, cytotoxic effector T cells, B cells, and other cell types, and it is involved in leukocyte recruitment associated with numerous inflammatory disorders [94].

Expression of both CX3CL1 expressed by vascular endothelial cells and other inflammatory cells and CX3CR1+ cytotoxic T cells and macrophages in muscle and in the lung affected by ILD was found in DM [95]. Serum level of CX3CL1 was higher in DM [95], and a positive correlation was found between CX3CL1 and CK level and Aa-DO_2_ [95] and anti-MDA5-Ab [96].

#### 3.3.3. CXCR3 Chemokines: CXCL10 or IP-10

CXCL10 or IFN-*γ*-induced protein 10 (IP-10) is a chemokine which acts through its receptor CXCR3. Th1 activation not only induces the production of IFN-*γ* and TNF-*α*, which stimulates lymphocytes, but also the production of other cell types (neutrophils, monocytes, endothelial cells, fibroblasts, thyrocytes, keratinocytes, etc.) leading to the secretion of CXCL10. It leads to a positive feedback loop which initiates and perpetuates the immune cascade. A high level of CXCL10 is considered to be a Th1 marker [97].

The serum level of CXCL10 was elevated in DM [63] and in DM/CADM with anti-MDA5 or anti-ARS Ab [62]. In this study, CXCL10 was higher in ILD compared to non-ILD. A positive correlation was found between CXCL10 and with the global VAS score [63, 65] and the pulmonary VAS but not with ferritin level [65]. Interestingly, CXCL10 could be used as a prognosis marker as levels at 2 weeks after treatment initiation were significantly higher in nonsurvivors [98]. However, serum CXCL10 level was not different between survivors and nonsurvivors in ILD-DM [98] and between RP-ILD and C-ILD [65].

#### 3.3.4. CCL2 or MCP1

CCL2 is a member of the CC chemokine family and is a chemotactic factor for monocytes/macrophages. Not only monocytes themselves but also astrocytic cells, endothelial cells, keratinocytes, mesangial cells, smooth muscle cells, microglial cells, and fibroblasts can produce CCL2 [99]. CCL2 is known as a downstream gene of the type I IFN pathway [100].

CCL2 has been shown to be associated with survival (the serum CCL2 level is higher in nonsurvivors in ILD-DM) [98]. In this study by Oda et al., the survival rate after 52 weeks was significantly lower in patients with a level of CCL2 of ≥900 pg/mL than in those with <900 pg/mL; serum CCL2 levels were significantly higher in the nonsurvivors than in the survival group. A positive correlation was found between the change of CCL2 (between first and second visit) and the change of PGA, muscle score, and extramuscular score [67]. CCL2 was shown to be strongly expressed in the muscle biopsy specimen near and within the vessels [47].

***To conclude, as cytokines, many chemokines seem to play a key role in the pathogenesis of DM recruiting inflammatory cells. However, some results although encouraging need to be validated in larger studies (CX3CL1).***

***CXCL8 has been found elevated in several studies, particularly during ILD and CADM, and correlated with the activity of the disease.***

***Similarly, CXCL10 and CCL2 are elevated in DM and CADM, are correlated with clinical activity, and could be used as treatment response and survival markers, respectively.***

### 3.4. Soluble Clusters of Differentiation

#### 3.4.1. Soluble CD163

CD163 is a glycosylated membrane protein that is expressed almost uniquely on monocytes and macrophages and more peculiarly on M2 macrophages. It is the macrophage scavenger receptor that takes up haptoglobin-hemoglobin complexes. Interestingly, in the context of DM, CD163 can be upregulated *in vitro* by glucocorticoids, IL-6, and IL-10 and downregulated by TNF-*α* and IFN-*γ* [101]. CD163 can be shed from the cell surface under several stimuli (i.e., LPS, immune complexes, and glucocorticoids) with a physiological role still unknown.

sCD163 was found elevated in DM compared to HD in several studies [102–105]. sCD163 was associated with ILD and ILD-related death in a study by Enomoto et al. [102]; it was not confirmed in a recent study by Kawasumi et al. [105]. The level of sCD163 seems to decrease after treatment.

#### 3.4.2. Soluble CD279 or sPDL1

Programmed death ligand 1 (PD-L1) is normally expressed on resting T cells, B cells, macrophages, and dendritic cells, whereas PD-L2 expression is expressed by macrophages and dendritic cells. Expression of PD-L1 is induced by inflammatory cytokines (IFN-*γ* or IL-10), and activation of PD-1 by its ligands suppresses T cell activation. Tumor cells overexpress PD-L1, which triggers T cell anergy or even death and thereby making the tumor cells capable of actively evading the immune system. It has been shown that a soluble form of PD-L1 can be detected in the sera of patients, which correlates with the amount of PD-L1-expressing cells. The expression of PD-L1 has been shown to negatively correlate with cancer prognosis [106].

In one study by Chen et al. [107], sPDL1 serum level was higher in DM without malignancies compared to that in SLE and HD. Interestingly, the serum level of sPDL1 was higher in new-onset cancer-related DM compared to DM without malignancies or stable cancers with DM. Unfortunately, sPDL1 could only distinguish new-onset cancer-related DM from DM without malignancies with a relatively poor sensitivity of 68%. This sensitivity was lower than the sensitivity of anti-TIF1-*γ* Ab.

*To conclude, soluble clusters are encouraging markers but they need further confirmation in larger studies.*

### 3.5. Complete Blood Count

#### 3.5.1. NLR

Neutrophil-to-lymphocyte ratio (NLR) is a simple parameter to easily assess the inflammatory status of a subject. It has proven its usefulness as a strong prognostic factor in several types of cancers or as a predictor and a marker of inflammatory or infectious pathologies.

NLR was found higher in DM compared to HD [108]. Several studies showed that NLR was higher in ILD compared to non-ILD [109–111]. Concerning clinical correlation, NLR was associated with global VAS score [110] and with overall mortality in all DM/CADM/PM [109] and in ILD-DM [111].

#### 3.5.2. Low-Density Granulocytes

DM and more particularly DM associated with ILD seems associated with the dysregulation of neutrophils and abnormal regulation of neutrophil extracellular traps (NETs). Similarly, with normal-density neutrophils, low-density granulocytes (LDGs) display pathogenic features and can also form NETs. LDGs can excessively secrete type I IFNs, TNF-*α*, and IFN-*γ*, which may induce more neutrophils to generate NETs in vivo. LDGs can also induce significant cytotoxicity for endothelial cells and disrupt the capacity of endothelial progenitor cells to differentiate into mature endothelial cells.

The percentage of the LDGs of DM patients was shown to be significantly higher than that of HD patients. DM patients with ILD had 2.7 times higher LDG percentages than did DM patients without ILD. LDG percentages positively correlated with MYOACT lung disease activity scores but not with other scores or clinical or biological parameters [112].

#### 3.5.3. Erythrocyte Sedimentation Rate (ESR)

Erythrocyte sedimentation rate is a routinely used laboratory marker reflecting acute-phase protein synthesis. It is thereby a surrogate marker of systemic inflammation.

Elevated ESR is reported to be of bad prognosis in dermatomyositis. In the 63 patients of a retrospective Chinese cohort, elevated ESR was an independent risk factor for in-hospital mortality. The patients died of three main causes: infection, ILD, and association of ILD and infection [113]. Go et al. [114] reported in a retrospective cohort that DM patients with baseline ESR ≥ 30 mm/h had significantly higher mortality, mainly related to respiratory failure. A persistently high ESR level was predictive of treatment resistance. The authors evoke a greater cytokine production in the context of ILD inducing an acute-phase response. In a case cohort study, an erythrocyte sedimentation rate higher than 35 mm/hr was strongly associated with the presence or the development of a malignancy [115]. Thus, despite heterogeneous data, baseline elevated ESR seems to be associated with ILD and mortality.

***To conclude, biomarkers derived from a complete blood count are useful and cheaper than many other new biomarkers. NLR and ESR, although nonspecific and associated with systemic inflammation, can be a precious tool to follow treatment response and to predict mortality.***

### 3.6. Other Serum Proteins

#### 3.6.1. CK

Creatine kinases (CKs) are cytosolic or mitochondrial enzymes with wide tissue distribution. Three isoenzymes are described. CK-MM is mainly distributed in striatal muscle and represents more than 95% of the total serum CKs in normal conditions.

It is a routinely used biomarker for myositis diagnosis and follow-up in DM. Biases are well known: physical exercise, drugs, and myocardial infarction can increase total CK levels.

Given that some subgroups of clinically amyopathic DM have worse prognosis due to ILD, it has been reported to have a prognostic value: normal or mild CPK level was associated with corticosteroid resistance [116, 117]. CKs were not assessed as a biomarker with DM-associated ILD.

#### 3.6.2. HSPA5

Heat shock 70 kDa protein 5 (HSPA5) is a member of the heat shock protein 70 (hsp70) family. HSPA5 is involved in the folding and assembly of proteins in the endoplasmic reticulum and may play a key role in the correct folding of proteins and degradation of misfolded proteins.

In one study, HSPA5 was significantly higher in DM compared to both HD and PM. Serum level decreased significantly after treatment but no correlation was studied with clinical parameters or prognosis [118].

#### 3.6.3. Troponin

Primary cardiac involvement in IIM and particularly DM is common and often subclinical and associated with poor prognosis. Both myocardial fibrosis and cardiovascular diseases are thought to take part in the process of cardiac involvement [119].

Erlacher et al. measured cardiac troponin T (cTnT), cardiac troponin I (cTnI), myosin heavy chains, myoglobin, creatine kinase (CK), and creatine kinase isoenzyme MB (CKMB) from 15 DM patients without any clinical evidence for acute cardiac affection. CKMB was increased in 51%, cTnT was increased in 41%, and cTnI was only elevated in 2.5% of the patients. There was no correlation between cTnI and other muscle or myocardial biomarkers or clinical activity [120].

Therefore, Hughes et al. recommend that, if elevated, the measurement of cTnT should be followed by the measurement of cTnI to confirm the cardiac involvement [119].

#### 3.6.4. Ferritin

Ferritin is the major intracellular iron storage protein in all organisms. TNF-*α* and IL-1*α* induce the expression of the H chain of ferritin in muscle cells and other cell types. Translation of ferritin is induced by IL-1*β*, IL-6, or TNF-*α* in the HepG2 hepatic cell line. Expression of ferritin is also regulated by hormones (thyroid and insulin), growth factors (insulin growth factor-1), second messengers, and hypoxia-ischemia and hyperoxia (nitric oxide).

Many studies have found an association between serum ferritin level and ILD during DM. Ferritin level is elevated in ILD-DM compared to non-ILD and also in RP-ILD compared to C-ILD [23, 48, 65, 121, 122]. Ferritin level was associated with clinical scores (global and pulmonary VAS) [65] and could be used as a treatment response prediction in anti-MDA5-Ab-positive patients as it was decreased only in survivors compared to nonsurvivors [23]. Interestingly, the ferritin level seems to be associated with early overall mortality [51, 111, 121, 123, 124] but data concerning late mortality are controversial [123, 125].

#### 3.6.5. Von Willebrand Factor

Von Willebrand factor (vWF) is a circulating glycoprotein that serves as a carrier for factor VIII in plasma. vWF is considered a circulating marker of endothelial cell activation. It has been reported that the vWF level is correlated with the activity of several diseases with vascular involvement and particularly juvenile DM.

In a study by Komiya et al. [126], serum vWF was elevated in the active group compared to HD and to inactive DM. In multivariate logistic regression, vWF was associated with fatigue, fever, and muscle weakness.

#### 3.6.6. SP-D

Surfactant protein D (SP-D) belongs to the collectin subgroup of the C-type lectin superfamily, which is produced and secreted by type II alveolar cells. Its serum level elevation could reflect lung damage, and it is reported in acute respiratory distress syndrome, IP, idiopathic pulmonary fibrosis, and IP associated with connective tissue disease.

SP-D serum level is higher in ILD-active DM compared to those with inactive or without IP [127–129]. However, SP-D levels at the diagnosis of active interstitial pneumonia failed to predict clinical course. Changes in serum level could be of bad prognosis during the first four weeks of therapy [127].

#### 3.6.7. KL-6

KL-6, a mucin-like high-molecular-weight glycoprotein, is strongly expressed in type II alveolar cells, and could similarly be a useful marker of lung damage. Serum KL-6 level is higher in DM with active IP, and early changes in serum level, similar to SP-D, were associated with poor prognosis [127]. KL-6 serum levels correlated with ILD activity in several retrospective [130] and prospective [129, 131] studies. Consistent findings were noted in other connective tissue diseases [132, 133].

***CK and troponin should be routinely measured in the care of DM patients for diagnosis and disease activity follow-up. Ferritin dosage is also suitable in these contexts and have prognostic value. KL-6 and SP-D appear to be promising biomarkers in DM-related ILD management.***

### 3.7. Virus Replication: Cytomegalovirus Reactivation

Cytomegalovirus (CMV) belongs to the herpesvirinae subfamily of herpesviridae that cause morbidity and mortality in immunocompromised patients.

In a retrospective cohort of autoimmune diseases, CMV reactivation with elevated viral load was significantly associated with the subgroup of PM/DM [134].

It is unknown whether CMV plays a pathogenetic role or whether CMV disease is an opportunistic infection. Indeed, lymphopenia was significantly associated with elevated viral load. It could be a causative factor for CMV reactivation or an early sign of CMV reactivation, as it is known that CMV can cause bone marrow suppression. In a retrospective case control study, CMV reactivation occurred regardless of the dosage and duration of corticosteroid administration, and no reactivation was observed in control patients (steroid-treated autoimmune diseases), questioning a contribution in the pathogenesis of DM [135]. Prospective trials are needed to assess the reliability of CMV load as a disease activity biomarker.

## 4. Conclusion

Serum biomarkers are useful tools emerging in the field of autoimmunity to help clinicians in the diagnosis and detection of visceral involvement and peculiar association of cancers in DM; such biomarkers will be more and more used to assess the response to treatment, predict the outcome, and finally help choose the appropriate treatment for the appropriate patient. This review highlights markers which may play a role in the pathogenesis of DM and can therefore be used to follow the disease. However, most of the results reported in this review should be validated in larger international studies as there are some discrepancies and clinical presentations that seem to be associated with ethnic origins.

MSAs enable disease classification and guide organ involvement and malignancy screening strategy. Concerning visceral involvement and clinical association of cancers, MSAs should be exhaustively searched during diagnosis; however, MSAs are poor diagnostic tools as half or more of DM patients do not exhibit MSAs. Concerning the prognosis of overall mortality and ILD-related mortality, the initial dosage of ferritin, IL-6, IL-18, NLR, SPD, and KL6 can be helpful. Concerning clinical association and follow-up of the treatments' response, anti-MDA5, anti-NXP-2, type I IFN, IL-18, IL-6, BAFF, ferritin, SPA/SPD, and KL6 can be used and repeated over time.

## Abbreviations

Aa-DO_2_: Alveolar–arterial gradient of oxygen
Ab: Antibody
APRIL: A proliferation-inducing ligand
AUC: Area under the curve
BAFF: B cell-activating factor
CADM: Clinical amyopathic dermatomyositis
CD: Cluster of differentiation
CDASI: Cutaneous disease severity index
cDM: Classical dermatomyositis
C-ILD: Chronic interstitial lung disease
CK: Creatine kinase
CKMB: Creatine kinase isoenzyme MB
CRP: C-reactive protein
cTnI: Cardiac troponin I
cTnT: Cardiac troponin T
DLCO: Diffusing capacity of the lung for carbon monoxide
DM: Dermatomyositis
ENMC: European Neuromuscular Center
HD: Healthy donors
HMGB1: High-mobility group protein 1
HR: Hazard ratio
HSPA5: Heat shock 70 kDa protein 5
IFN: Interferon
IIM: Idiopathic inflammatory myopathy
IL: Interleukin
ILD: Interstitial lung disease
IP-10: IFN-*γ*-induced protein 10
KL-6: Krebs von den Lungen-6
LDGs: Low-density granulocytes
LIGHT: Homologous to lymphotoxin, exhibits inducible expression and competes with HSV glycoprotein D for binding to herpesvirus entry mediator, a receptor expressed on T lymphocytes
MAA: Myositis-associated antibody
MCP: Monocyte chemoattractant protein
MDA5: Melanoma differentiation-associated gene 5
MHC: Major histocompatibility complex
MITAX: Myositis intention to treat activity index
MSA: Myositis-specific antibody
NETs: Neutrophil extracellular traps
NK: Natural killer
NLR: Nod-like receptor
NXP-2: Nuclear matrix protein 2
PDL: Programmed death ligand
PGA: Physician global assessment
PM: Polymyositis
RIG: Retinoic acid-inducible gene
RNA: Ribonucleic acid
RP-ILD: Rapidly progressive interstitial lung disease
RT-PCR: Reverse transcription-polymerase chain reaction
SAE: Small ubiquitin-like modifier-activating enzyme
SLE: Systemic lupus erythematosus
SP-D: Surfactant protein D
TGF: Transforming growth factor
TIF: Transcription
TLR: Toll-like receptor
TNF: Tumor necrosis factor
VAS: Visual analogue scale
vWF: Von Willebrand factor.
